# Delayed Ego Strength Development in Opioid Dependent Adolescents and Young Adults

**DOI:** 10.1155/2015/879794

**Published:** 2015-11-17

**Authors:** Benjamin A. Abramoff, Hannah L. H. Lange, Steven C. Matson, Casey B. Cottrill, Jeffrey A. Bridge, Mahmoud Abdel-Rasoul, Andrea E. Bonny

**Affiliations:** ^1^Department of Physical Medicine and Rehabilitation, Emory University, Atlanta, GA 30322, USA; ^2^The Research Institute at Nationwide Children's Hospital, Columbus, OH 43205, USA; ^3^The Ohio State University, Columbus, OH 43210, USA; ^4^Nationwide Children's Hospital, Columbus, OH 43205, USA

## Abstract

*Objective*. To evaluate ego strengths, in the context of Erikson's framework, among adolescents and young adults diagnosed with opioid dependence as compared to non-drug using youth.* Methods*. Opioid dependent (*n* = 51) and non-drug using control (*n* = 31) youth completed the self-administered Psychosocial Inventory of Ego Strengths (PIES). The PIES assesses development in the framework of Erikson's ego strength stages. Multivariate linear regression modeling assessed the independent association of the primary covariate (opioid dependent versus control) as well as potential confounding variables (e.g., psychiatric comorbidities, intelligence) with total PIES score.* Results*. Mean total PIES score was significantly lower in opioid dependent youth (231.65 ± 30.39 opioid dependent versus 270.67 ± 30.06 control; *p* < 0.01). Evaluation of the PIES subscores found significant (*p* < 0.05) delays in all ego strength areas (hope, will, purpose, competence, fidelity, love, care, and wisdom). When adjusting for potential confounders, opioid dependence remained a significant (*p* < 0.001) independent predictor of total PIES score.* Conclusion*. Adolescents with opioid dependence demonstrated significant delays in ego strength development. A treatment approach acknowledging this delay may be needed in the counseling and treatment of adolescents with opioid dependence.

## 1. Introduction

Opioid misuse and subsequent medical complications have reached epidemic levels among adolescents and young adults in the United States [1–6]. According to national surveys, over 10% of high school seniors report lifetime misuse of prescription opioids [7]. As this epidemic continues to unfold, a deeper understanding of those affected will be necessary to inform appropriate interventions and treatments.

Opioid misuse is of special concern in adolescent and young adult populations. Prior research has found that substance abuse during adolescence is associated with problem behaviors including poor school performance [8], juvenile delinquency [9], and risky sexual practices [10]. Additionally, nonmedical use of prescription opioids during adolescence is associated with substance misuse and dependence in adulthood [11], meaning that youth who misuse opioids may accumulate greater exposure throughout the lifespan. As the brain undergoes significant physiologic changes during adolescence, opioid misuse during this phase of life can have lasting effects on neurodevelopment [12, 13]. However, the long-term psychosocial consequences of opioid misuse during adolescence are not well understood.

The period of adolescence and young adulthood is marked by the psychosocial task of identity formation [14–17]. As described in the works of Erikson, identity is a personal sense of self, consisting of the sum of one's lived experiences [14]. Identity development includes both intrapersonal self-definition and interpersonal relationships [14]. A mature, coherent identity facilitates overall well-being and successful transition to adulthood [18]. While initial substance use tends to occur during identity formation, the relationship between substance misuse and psychosocial maturity is not clear [19]. Though higher levels of psychosocial maturity may be protective against health risk behaviors such as substance abuse [20], substance abuse at this critical time may in fact delay identity formation.

The framework of ego strengths may be useful in delineating identity formation differences between adolescents who do and do not misuse opioids. As defined by Erikson, ego strengths comprise vital characteristics central to maturation [21, 22]. Ascendance of each ego strength is thought to indicate successful resolution of a corresponding psychosocial stage [23]. Thus, ego strengths can be considered indicators of psychosocial health at a given stage of development [23]. All ego strengths are present in some form throughout the lifespan; strengths arise after achievement of others and, in turn, inform those that came before [23]. Together, ego strengths are indicative of general well-being, resiliency, and psychosocial maturity [23, 24].

Although a number of tools have been created to assess specific areas of adolescent development, we know of no previous study that has attempted to evaluate a global measure of adolescent psychosocial maturity in a population of opioid dependent adolescents. The objective of this study was to evaluate ego strengths, in the context of Erikson's framework, among adolescents and young adults diagnosed with opioid dependence as compared to non-drug using youth.

## 2. Methods

### 2.1. Study Population

Opioid dependent participants consisted of adolescents and young adults, age 16–22, who presented to the Medication Assisted Treatment for Addiction (MATA) program at Nationwide Children's Hospital between October 2012 and May 2013. A confirmed diagnosis of opioid dependence using DSM IV-R criteria was required for entrance into the MATA program and study participation [25]. The MATA program, which has previously been described in detail, prescribes buprenorphine/naloxone to opioid dependent adolescents and young adults engaged in a substance abuse treatment program while concurrently managing other medical and mental health conditions [26]. Control participants consisted of non-drug using youth, age 16–22 years, recruited from a local shopping mall and through institutional e-mails directed at employees and their families.

Exclusion criteria for both opioid dependent and control subjects included inability to comprehend written English or limited cognitive abilities preventing completion of study assessments. Control subjects were excluded from analysis for any of the following: (1) any opioid misuse in the past thirty days; (2) lifetime nonprescription opioid use of three or more times; (3) lifetime use of any other illicit substance of three or more times; or (4) ever having used a needle to inject any illicit substance.

The study protocol was reviewed and approved by the Institutional Review Board (IRB) of the participating institution. Since opioid dependent adolescents can seek substance abuse treatment without parental consent, the IRB requested differing consent procedures for opioid dependent and control participants; opioid dependent participants provided verbal consent while control participants (and one legal guardian of participants <18 years of age) provided signed consent.

During regularly scheduled MATA clinic visits, eligible opioid dependent patients were briefly told about the study by their treatment team. If a patient expressed interest, research staff explained the study procedures in detail. An informational study pamphlet was provided and verbal consent was obtained from all opioid dependent participants. After consent was obtained, participants were given the option of completing the study during their scheduled MATA program visit or returning for a separate study appointment in the clinical research unit (CRU) of the participating institution. As such, 51 opioid dependent adolescents enrolled in the study.

To identify potential control participants, two recruitment strategies were employed. A recruitment stand was set up at a local shopping mall and institutional e-mails were sent to employees soliciting interested participants. These recruitment strategies were intended to access populations that would be similar to the MATA participants; the shopping mall was located in the zip code where the majority of MATA patients resided. Once identified, potential control participants met with research staff to have the study explained in detail. Written informed consent from one legal guardian and subject assent were required of control participants below 18 years of age. Written informed consent was required of control subjects 18 years of age and older. After consent was obtained, control subjects were given the option to immediately participate in the study in a private area of the mall or to schedule a separate study appointment in the CRU. All control subjects recruited by institutional e-mail completed their study visit in the CRU. Of the 45 control participants who were screened, 14 were excluded based on prior opioid and/or other drugs' use, leaving 31 eligible controls.

### 2.2. Measures

Following consent procedures, participants self-administered the Psychosocial Inventory of Ego Strengths (PIES), a modified Youth Risk Behavior Survey (YRBS), and the Symptom Checklist-90 (SCL). Trained study staff administered the Kaufman Brief Intelligence Test, Second Edition (K-Bit), to each subject. All measures were collected and managed on secured laptop computers using REDCap electronic data capture tools hosted at Nationwide Children's Hospital [27].

The Psychosocial Inventory of Ego Strengths (PIES) was developed by Markstrom et al. to assess degree of ego strength, an indicator of psychosocial wellness in the Eriksonian framework [23]. The PIES consists of 64 items devised by Markstrom et al. and assesses adolescent development in the framework of Erikson's eight stages of psychosocial development [23]. All 64 items are summed to derive a total ego strength score. Eight subscores are derived from items specific to each ego strength: hope (e.g.,* “When I think about the future I feel optimistic.”*); will (e.g.,* “If there is something I choose to do, I am determined to do it.”*); purpose (e.g.,* “I am able to set realistic goals for myself.”*); competence (e.g.,* “I know I have skills to carry out various tasks and responsibilities important to me.”*); fidelity (e.g.,* “I believe in being true to myself and others.”*); love (e.g.,* “When I love someone I can accept that they might want to pursue some interests without me.”*); care (e.g.,* “When I see someone with a need, I help in whatever way I am able.”*); and wisdom (e.g.,* “I can accept the fact that I've made mistakes in my life.”*). Respondents answer each item on a five-point scale ranging from 1, “does not describe me well,” to 5, “describes me very well.” Higher scores on the PIES have been found to be associated with other measures of adolescent psychosocial development including self-esteem, internal locus of control, perspective taking, empathetic concern, and positive coping skills [23, 24]. Reliability of the PIES has been demonstrated in prior research among undergraduate students (alpha 0.93 for the combined items and ranging from 0.69 to 0.86 for the eight subscales) and high school students (alpha 0.94 for the combined items and ranging from 0.60 to 0.83 for the eight subscales) [23, 24]. High scores on all PIES subscales are indicative of good psychosocial health and psychosocial maturity [23].

The YRBS is a nationally representative survey administered biannually to US students in grades 9–12 by the Centers for Disease Control and Prevention which asks about a broad spectrum of health risk behaviors [28, 29]. For the current study, all YRBS questions pertaining to alcohol, tobacco, and illicit drugs were utilized to assess drug use patterns (e.g.,* “During your life, how many times have you taken a prescription pain drug (such as OxyContin, Percocet, Vicodin, codeine), without a doctor's prescription?”* Responses: 0 times, 1 or 2 times, 3 to 9 times, 10 to 19 times, 20 to 39 times, and 40 or more times). YRBS questions were used to ensure that opioid dependent and control subjects differed with regard to substance misuse and that control subjects were indeed eligible.

The SCL-90 was used to assess possible psychiatric comorbidity as we anticipated that psychiatric comorbidity could be related to both ego strength development and/or opioid misuse. The SCL-90 is a self-administered 90-question survey that assesses current, point-in-time psychological symptom status in subjects 13 years of age and older (available from http://www.pearsonclinical.com). The SCL-90 is often used in screening and psychiatric research [30]. The SCL-90 assesses nine symptom dimensions: somatization, psychoticism, paranoid ideation, hostility, phobic anxiety, depression, anxiety, obsessive-compulsive symptoms, and interpersonal sensitivity. Respondents are asked to consider,* “In the previous week, how much were you bothered by”* symptoms such as* “nervousness or shakiness inside,” “feeling lonely,” “temper outbursts that you could not control,”* and* “feeling hopeless about the future*.*”* Respondents rate each item on a five-point scale of distress ranging from 0 (not at all) to 4 (extremely). A Global Severity Index (GSI), designed to give an overall evaluation of a subject's mental health, is calculated in addition to scores for each of the nine domains. Previous research has shown the SCL-90 to be clinically useful for diagnosis of anxiety and mood disorders in substance abuse populations [31].

In order to better control for additional factors which could contribute to measured psychosocial maturity, intelligence was assessed utilizing the K-Bit. The K-Bit is a brief (15–30 minutes) test which has been nationally standardized for ages 4 through 90. The test consists of two verbal intelligence subtests and one nonverbal intelligence subtest, which generate a total intelligence score. For verbal intelligence, the examiner presents a set of pictures and says a vocabulary word (e.g.,* “cleanse,” “athletic”*), asks a general knowledge question (e.g.,* “what helps you breathe”*), or says a verbal riddle (*“What is made of rubber, is usually pink, and gets rid of mistakes?”*). The examinee then chooses the appropriate picture. For nonverbal intelligence, the examinee is asked to find a relationship or rule in a set of pictures or patterns. The K-Bit is recommended for screening purposes in a variety of settings [32].

### 2.3. Analyses

The primary outcome measure was overall ego strength development as reflected by the total PIES score. Secondary outcome measures included scores on each of the eight PIES individual ego strength subscales. Three opioid dependent participants and 2 control participants were excluded from the analysis of the main outcome because they did not answer the PIES questionnaire or SCL-90.

Chi-squared or Fisher's exact tests (categorical variables) and Student's *t*-tests (continuous variables) were used to compare demographic and clinical characteristics of opioid dependent and control subjects. A multivariate linear regression model was constructed to assess the independent association of study group with total PIES score, adjusting for potential confounders. Potential confounders included age; gender; race; insurance status (public, private, or none); place of residence (rural, suburban, or urban); maternal education; paternal education; family history of drug abuse; past month alcohol and tobacco use; SCL GSI; and K-Bit verbal, nonverbal, and total intelligence scores. Two-tailed *p* values of less than 0.05 were used as the level of statistical significance. Data analyses were conducted using SAS Statistical Software (Version 9.3; SAS Institute, 2011).

## 3. Results

Significant differences were found between opioid dependent (*n* = 51) and control (*n* = 31) participants with regard to age, insurance status, family history of drug abuse, and past month tobacco use (Table 1). Mean age was 19.3 ± 1.4 years in the opioid dependent group and 18.3 ± 1.7 years in the control group. In general, opioid dependent participants were more likely to have Medicaid insurance, report family history of drug abuse, and have used tobacco on at least 10 days during the past month. No statistically significant differences were observed between opioid dependent and control participants with regard to gender, race, place of residence, parental education, and past month alcohol use.

As shown in Table 1, opioid dependent participants demonstrated significantly higher levels of psychological distress as measured by the mean SCL GSI score (opioid dependent 1.02 ± 0.69 versus control 0.53 ± 0.44, *p* < 0.01). Higher scores were seen across all SCL psychological domains among opioid dependent youth as compared to non-drug using controls. These differences were statistically significant in all domains with the exception of psychoticism and interpersonal sensitivity. Control participants had significantly higher mean intelligence as measured by the total K-Bit score (control 94.2 ± 10.1 versus opioid dependent 87.1 ± 11.7, *p* < 0.05) and verbal and nonverbal intelligence subscores.

Evaluation of the unadjusted associations of PIES total and subscale scores and study group found significant differences between opioid dependent and control participants (Table 2). A 39-point difference in total mean PIES score was evident between opioid dependent and control subjects. In addition, opioid dependent youth exhibited significantly lower scores in every ego strength domain.

In multivariable modeling, including adjustment for all potential confounders, study group (opioid dependent versus control) remained a significant predictor of total PIES score (Table 3). On average, opioid dependent participants scored 26.42 points lower (95% CI: 40.36 to 12.49) on the total PIES relative to controls. In addition, age, SCL GSI score, and total intelligence score remained independently predictive of total PIES score.

## 4. Discussion

The purpose of our study was to conduct a systematic, global evaluation of ego strength maturation, in the context of Erickson's framework, among adolescents and young adults diagnosed with opioid dependence as compared to non-drug using youth. Ego strengths are related to coping skills that help to manage life's challenges [23]. As such, they are of interest in opioid dependent populations and may have implications for treatment and recovery. For example, hope is reflective of confidence and optimism about life, people, oneself, and the future. Will is reflective of one's ability to exercise free choice, self-restraint, and self-control. Purpose implies a form of courage to pursue goals in spite of fear or guilt. Fidelity represents ideological commitment and sense of personal identity, typically achieved during adolescence and early adulthood [23]. Taken together, ego strengths indicate psychosocial maturity and personal resilience [24].

Among our study cohort, opioid dependent adolescents and young adults scored significantly lower on both a global measure of ego strength maturation and individual ego strength domains as compared to non-drug using youth. The noted differences in PIES scores were not due to deficiencies in any one, individual, ego strength but rather were found to exist across all ego strength subscales. These differences were still observed after controlling for age, psychiatric comorbidity, gender, and intelligence.

The magnitude of difference in PIES score found among our opioid dependent and control study participants was much larger than that attributed to gender or age in previous studies. Markstrom and Marshall administered the PIES to university and high school students in the United States [24]. They found differences between male and female respondents in mean total PIES score with females generally scoring higher. The average difference in total PIES score attributed to gender in their study cohort was 10.8. In addition, Markstrom and Marshall identified differences in total PIES scores between high school and university students, with high school students generally scoring lower. The average difference in total PIES score between these high school and university students was 5.6. Among our study sample, after adjusting for age, gender, psychological distress, and intelligence, the average group difference between opioid dependent and non-drug using youth on total PIES score was 26.42. Given the magnitude of difference found between our study groups, the association between opioid dependence and ego strength maturation appears to be even stronger than that previously attributed to age or gender.

### 4.1. Limitations

Despite attempts to control for major confounding variables, ego strength development is likely impacted by additional factors unmeasured in the current study such as early childhood experiences, presence of chronic medical conditions, and history of trauma. In addition, the cross-sectional nature of the current data precludes any inferences about causality or potential impact on treatment outcomes. The current level of evidence precludes one from concluding whether ego strength maturation impacts substance use risk or whether substance use results in maturation delays. Longitudinal data is needed to fully define the relationship between ego strength and drug dependence and to determine the nature of causality if present. The association between opioid dependence and ego strength maturation is likely a complicated one, encompassing both individual and environmental factors. For example, family history of drug abuse was much more prevalent in the opioid dependent group. Although family history of drug abuse was not independently associated with PIES score in multivariate modeling, this factor likely contributed to the overall impact of study group.

## 5. Conclusions

This study found that opioid dependent youth have significantly lower ego strength development than non-drug using peers even when controlling for age, gender, psychological distress, and intelligence. Ego strength deficits were evident among all of the individual ego strength domains. This delayed psychosocial maturity is an important consideration in the treatment of opioid dependence among adolescents and young adults. Erikson argued that successful therapy should result in not only diminished symptoms, but also increased ego strengths [23]. Thus, assessment of a patient's ego strength in conjunction with overall character can serve to guide effective treatment [33].

Psychosocial models of recovery place emphasis on redefining identity and striving toward an “ideal self” [34]. This sense of self and purpose appears to be delayed in opioid dependent youth. As adolescents of differing developmental stages likely respond differently to intervention efforts, customizing treatment programs according to level of maturity may serve to maximize efficacy [20]. Evaluation of ego strengths among opioid dependent adolescents and young adults may serve to inform tailored intervention efforts to effectively address the growing opioid misuse epidemic.

## Figures and Tables

**Table 1:** Baseline subject characteristics by study group (opioid dependent versus control).

Characteristic	Opioid dependent (*n* = 51)	Control (*n* = 31)	*p* value
Age (years)			<0.01
Mean (SD)	19.3 (1.4)	18.3 (1.7)	
Gender, *n* (%)			0.78
Female	33 (64.7)	21 (67.7)	
Male	18 (35.3)	10 (32.3)	
Race, *n* (%)			0.05
White	51 (100.0)	28 (90.3)	
Black	0 (0.0)	2 (6.5)	
Not specified	0 (0.0)	1 (3.2)	
Insurance status, *n* (%)			<0.01
Private	14 (27.5)	9 (29.0)	
Medicaid	28 (54.9)	5 (16.1)	
No insurance	5 (9.8)	3 (9.7)	
Unknown	4 (7.8)	14 (45.2)	
Place of residence, *n* (%)			0.40
Rural	19 (27.5)	12 (38.7)	
Suburban	17 (33.3)	14 (45.2)	
Urban	11 (21.6)	5 (16.1)	
Maternal education, *n* (%)			0.95
Less than high school	9 (17.7)	4 (12.9)	
High school degree or equivalent	17 (33.3)	10 (32.3)	
Some college or greater	22 (43.1)	15 (48.4)	
Unknown	3 (5.9)	2 (6.4)	
Paternal education, *n* (%)			0.16
Less than high school	10 (19.6)	2 (6.5)	
High school degree or equivalent	24 (47.1)	12 (38.7)	
Some college or greater	12 (23.5)	14 (45.2)	
Unknown	5 (9.8)	3 (9.7)	
Family history of drug abuse, *n* (%)			<0.01
Yes	46 (90.2)	15 (48.4)	
No	5 (9.8)	16 (51.6)	
Past month alcohol use, *n* (%)			0.88
None	33 (66.0)	19 (61.3)	
1 or 2 days	12 (24.0)	10 (32.3)	
3–9 days	3 (6.0)	1 (3.2)	
10 or more days	2 (4.0)	1 (3.2)	
Past month tobacco use, *n* (%)			<0.01
<10 days	3 (6.1)	27 (87.1)	
10 or more days	46 (93.9)	4 (12.9)	
SCL-90 scores^1^, mean (SD)			
Somatization	1.09 (0.80)	0.50 (0.40)	<0.01
Psychoticism	0.57 (0.51)	0.38 (0.45)	0.09
Paranoid ideation	1.09 (0.86)	0.56 (0.71)	<0.01
Hostility	1.19 (0.95)	0.65 (0.79)	<0.01
Phobic anxiety	0.79 (0.82)	0.25 (0.40)	<0.01
Depression	1.23 (0.81)	0.59 (0.47)	<0.01
Anxiety	0.90 (0.79)	0.34 (0.37)	<0.01
Obsessive-compulsive symptoms	1.22 (0.79)	0.69 (0.69)	<0.01
Interpersonal sensitivity	0.95 (0.81)	0.68 (0.74)	0.15
Other	1.16 (0.78)	0.65 (0.55)	<0.01
Global Severity Index	1.02 (0.69)	0.53 (0.44)	<0.01
Intelligence^2^			
Total	87.1 (11.7)	94.2 (10.1)	<0.01
Verbal	88.3 (9.7)	93.4 (9.5)	0.02
Nonverbal	89.1 (14.8)	96.2 (11.1)	0.02

^1^Symptom Checklist-90, self-report psychometric instrument.

^2^Total intelligence measured by the Kaufman Brief Intelligence Test, Second Edition.

**Table 2:** Unadjusted associations^1^  between Psychological Inventory of Ego Strength (PIES) total and subscale scores and study group (opioid dependent versus control).

Mean (SD)	Opioid dependent (*n* = 49)	Control (*n* = 31)	*p* value
Total PIES score	231.65 (30.39)	270.67 (30.06)	<0.01
Hope	27.47 (6.16)	33.51 (4.73)	<0.01
Will	28.24 (5.13)	34.27 (4.39)	<0.01
Purpose	28.87 (4.85)	33.85 (4.72)	<0.01
Competence	28.69 (5.00)	33.92 (4.79)	<0.01
Fidelity	30.49 (4.29)	34.23 (4.28)	<0.01
Love	31.03 (3.77)	33.50 (5.16)	0.02
Care	30.44 (5.28)	34.76 (4.36)	<0.01
Wisdom	26.42 (5.63)	32.62 (4.49)	<0.01

^1^Unadjusted Student's *t*-test.

**Table 3:** Multivariate linear regression model predicting total Psychological Inventory of Ego Strength (PIES) score.

Predictor	Model estimate	95% confidence limits	*p* value
Model intercept	150.30	65.96 to 234.65	<0.001

Opioid dependent group Control group	−26.42 Reference	−40.36 to −12.49 —	<0.001 —

SCL_GSI^1^	−27.96	−37.93 to −17.99	<0.001

Intelligence^2^	0.62	0.11 to 1.13	0.018

Age	4.37	0.45 to 8.30	0.030

Female gender Male gender	−4.34 Reference	−17.94 to 9.26 —	0.527 —

^1^Symptom Checklist-90, Global Severity Index.

^2^Total intelligence measured by the Kaufman Brief Intelligence Test, Second Edition.
